# Suicide risk in primary care patients diagnosed with a personality disorder: a nested case control study

**DOI:** 10.1186/s12875-016-0479-y

**Published:** 2016-08-05

**Authors:** Michael Doyle, David While, Pearl L. H. Mok, Kirsten Windfuhr, Darren M. Ashcroft, Evangelos Kontopantelis, Carolyn A. Chew-Graham, Louis Appleby, Jenny Shaw, Roger T. Webb

**Affiliations:** 1 Centre for Mental Health and Safety, Institute of Brain, Behaviour and Mental Health, University of Manchester, Manchester, England; 2 South West Yorkshire Partnership NHS Foundation Trust, Wakefield, England; 3 Centre for Pharmacoepidemiology and Drug Safety, Manchester Pharmacy School, University of Manchester, Manchester, England; 4 NIHR Greater Manchester Primary Care Patient Safety Translational Research Centre, University of Manchester, Manchester, England; 5 The Farr Institute for Health Informatics Research, University of Manchester, Manchester, England; 6 NIHR School for Primary Care Research, University of Manchester, Manchester, England; 7 Research Institute, Primary Care and Health Sciences, Keele University, Keele, England; 8 West Midlands Collaboration for Leadership in Applied Health Research and Care (CLAHRC), Keele, England

**Keywords:** Personality disorder, Borderline personality disorder, Suicide, Alcohol misuse, Mental illness, Primary care, General practitioners, Clinical Practice Research Datalink

## Abstract

**Background:**

Personality disorder (PD) is associated with elevated suicide risk, but the level of risk in primary care settings is unknown. We assessed whether PD among primary care patients is linked with a greater elevation in risk as compared with other psychiatric diagnoses, and whether the association is modified by gender, age, type of PD, and comorbid alcohol misuse.

**Methods:**

Using data from the UK Clinical Practice Research Datalink, 2384 suicides were matched to 46,899 living controls by gender, age, and registered practice. Prevalence of PD, other mental disorders, and alcohol misuse was calculated for cases and controls separately and conditional logistic regression models were used to estimate exposure odds ratios. We also fitted gender interaction terms and formally tested their significance, and estimated gender age-specific effects.

**Results:**

We found a 20-fold increase in suicide risk for patients with PD versus no recorded psychiatric disorder, and a four-fold increase versus all other psychiatric illnesses combined. Borderline PD and PD with comorbid alcohol misuse were associated with a 37- and 45-fold increased risk, respectively, compared with those with no psychiatric disorders. Relative risks were higher for female than for male patients with PD. Significant risks associated with PD diagnosis were identified across all age ranges, although the greatest elevations were in the younger age ranges, 16–39 years.

**Conclusions:**

The large elevation in suicide risk among patients diagnosed with PD and comorbid alcohol misuse is a particular concern. GPs have a potentially key role to play in intervening with patients diagnosed with PD, particularly in the presence of comorbid alcohol misuse, which may help reduce suicide risk. This would mean working with specialist care, agreed clinical pathways and availability of services for comorbidities such as alcohol misuse, as well as opportunities for GPs to develop specific clinical skills.

## Background

Over 5,000 suicides occur annually in England and Wales, and suicide is the leading cause of death for 20–34 year olds [1]. A diagnosis of personality disorder (PD), in particular borderline PD, has been found to be strongly linked with elevated suicide risk [2–5]. For example, it has been reported that PD was associated with a seven-fold increase in suicide risk compared with those in the general population with no mental illnesses [5]. In fact, persons with PD are amongst those at greatest risk of suicide when compared to patients from other psychiatric diagnostic groups [6–8], while suicidal and self-injurious behaviour are seen as defining features of borderline PD [9]. It has been estimated that at least three-quarters of people with borderline PD have had at least one suicide attempt, and the risk of completed suicide amongst these patients may be as high as 10 % [2]. Patients with borderline PD are also found to have earlier onset of suicidal behaviour than those with depression only [2, 10]. Furthermore, individuals with PD have a raised prevalence of alcohol use disorders [11–13]. A study of over 40,000 individuals from the US general population has found that 42 % of those with a PD diagnosis had comorbid alcohol dependence compared with 13 % of those without PD [13].

It has been reported that PD is associated with frequent attendance to general practice and fewer referrals to secondary care [14], which in the UK, includes specialist mental health inpatient and outpatient services. Previous studies on suicidal behaviour amongst patients with PD were, however, mostly conducted in secondary care settings while surprisingly little is known about suicide risk among people with PD who present to primary care. Studies in the UK have also found that only 30 % of people who died by suicide had contact with mental health services in the preceding year [15], whereas 63 % contacted their General Practitioner (GP) in the same time period [16]. This suggests that GPs have a very important role in suicide prevention and better knowledge of suicide risk amongst primary care patients diagnosed with PD is needed.

In this study, we addressed the following research questions: 1) Is PD among primary care patients linked with a greater elevation in risk of suicide as compared to patients with no history of mental illness and to patients diagnosed with other psychiatric diagnoses? 2) Are these relative risks modified by gender and age? 3) Are patients with borderline PD at higher risk compared to those with all other types of PD? (4) Does risk associated with PD increase with comorbid alcohol misuse?

## Methods

### Data source: the Clinical Practice Research Datalink (CPRD)

In the UK, over 98 % of the population are registered with a general practice, with the clinical team providing primary care and access to most other services from the National Health Service (NHS) [17]. Even where the patient leaves a family practice and joins another, their GP record follows them, providing a continuous care record. Our data source was the UK Clinical Practice Research Datalink (CPRD), which is one of the largest population-based, longitudinal, primary care databases in the world (www.cprd.com). The CPRD has provided anonymised primary care records for public health and epidemiological research since 1987. It was initially established in London as the Value Added Medical Products (VAMP) ‘research bank’, which expanded across the UK to become the General Practice Research Database (GPRD) in 1993, and was developed further as the CPRD in 2012. It came into being because of a need to develop good quality IT computer systems for general practices in an era when most practices were still wholly reliant on paper records. All practices in the CPRD use Vision software rather than other general practice computing systems, and have consented to share anonymous data for academic research purposes. Around 7 % of the UK population is now represented in the CPRD [17]. Although practices were not selected according to any scientific sampling strategy, a fortuitous by-product of the largely ad hoc processes via which practices have contributed data to the Datalink is that it is broadly representative of the UK primary care patient population by basic demographics such as age, gender and ethnicity [17].

All consultations for registered patients in participating practices are recorded in the CPRD, with comprehensive and detailed clinical coding - the ‘Read’ codes - for symptoms, diagnoses, treatment (including prescribed medication), and referral to other forms of NHS care and to other health care providers. The September 2010 version of the CPRD we analysed contained approximately 10.6 million complete patient records. Before our study dataset was created, the Independent Scientific Advisory Committee of the CPRD granted approval. Consent from individual patients is not required to conduct CPRD-based studies.

To our knowledge there have been no published validation studies of personality disorder or alcohol misuse diagnoses in the CPRD. However, two systematic reviews have reported overall high levels of diagnostic validity in the CPRD and good levels of agreement with other routinely collected data sources [18, 19]. A generic limitation of the CPRD is that diagnostic behaviour varies considerably between GPs and between general practices. Different doctors may apply varying clinical Read codes for exactly the same condition, whilst some may enter free-text information instead of clinical coding. The Quality and Outcomes Framework was introduced in 2004, and recent evidence indicates that this national quality of care incentivisation initiative has produced a marked improvement in diagnostic accuracy and completeness for some chronic conditions during recent years in the CPRD [17].

The potential of this data source has been further enhanced by recent implementation of linkage to national mortality records. In 2008, complete prospective and historic linkage to national mortality registration data was implemented via the Office for National Statistics (ONS). Data linkage between CPRD and ONS is only available for English practices that have consented to participate in the linkage scheme. These linkages cover approximately 75 % of the contributing CPRD practices in England; the equivalent procedure has not yet been implemented for CPRD practices in the other UK nations: Scotland, Wales and Northern Ireland [17]. Therefore, our case-control study was nested within a subset of the whole CPRD. We included adult suicides if the individual died between January 1st 2002 and December 31st 2011, and had at least a complete year of “up-to-standard” CPRD clinical data prior to the individual’s index-date (death). This quality criterion was also applied in selecting the matched living controls.

### Suicide case definition

In the UK, most unnatural deaths of undetermined cause (or ‘open verdicts’) among adults are considered likely to be suicides [20]. To reduce false-negative misclassification, and in line with standard practice for conducting epidemiological studies of suicide in the UK, our case definition included these open verdicts [21]. The following ICD-10 codes were used: X60-84, Y10-Y34 (excluding Y33.9; ie deaths with adjourned inquests that are mostly deemed subsequently to be homicides), Y87.0 and Y87.2.

### Identifying PD, other mental disorders and comorbid alcohol misuse

To delineate diagnoses of PD we searched the textual descriptions of the clinical Read codes for terms that included: ‘personality’, ‘psychopath’ or ‘sociopath’. We examined the relevant Read codes descriptors and reached a general consensus that we would define patients as having a PD if they had a diagnosis that included a sub-string of either ‘personality disorder’ or ‘psychopathic disorder’ to ensure that we had a clinically relevant sample with a PD diagnosis. We then identified an additional subgroup of ‘borderline PD: diagnosis of borderline or unstable personality disorder’. The process used to delineate diagnosis of the disorders was similar to that described in Olier et al. [22].

We also constructed two additional medical definitions to identify patients with: (1) Any diagnosed mental health disorder: all Read codes beginning with the letter ‘E’, ie a diagnosis of ‘Mental Disorders’ (please note that we excluded those patients coded for signs and symptoms of mental illness only but without an ‘E…’ diagnostic code); (2) Comorbid alcohol misuse: all code descriptions containing “alcohol” were extracted. Two senior clinicians (co-author and consultant psychiatrist: JS; first author and forensic mental health nurse consultant: MD) then independently identified and agreed a list of codes that indicated alcohol problems using a RAG (Red/Amber/Green) agreement system, where Red was defined as a definite clinically significant alcohol misuse problem; Amber, possible problem and Green no problem. Independent ratings were reviewed and a consensus on Red, definite clinically significant alcohol misuse, was established. We opted not to investigate co-morbid PD and illicit drug use due to suspected classification issues, and because the number of suicide cases with such co-morbidity was expected to be very small in the CPRD.

The diagnoses of PD, any mental health disorder and the alcohol misuse definition are defined as ‘lifetime’ definitions in the sense that they are recorded at some point in their CPRD GP clinical records [23]. The codes contained in the Read ‘E’ category cover every type of diagnosable mental illness across the full spectrum of psychopathology, irrespective of levels of severity and chronicity. In their meta-analysis of suicide risk in persons with a mental disorder, Harris and Barraclough reported that virtually all mental disorders, except for intellectual disability and dementia, are associated with an increased risk of suicide [24]. We therefore combined all categories of mental illness other than personality disorders and analysed them together as a single category. In the UK, general practitioners typically diagnose and treat less severe disorders such as depression, anxiety and stress, whereas more series conditions such as psychotic disorders and PDs will be mostly treated by psychiatrists in inpatient or outpatient facilities. The great majority of persons with a diagnosed mental disorder in the study dataset were diagnosed with depression and/or anxiety disorders, and this applied to people who died by suicide as well as the living control patients. Mental illness diagnoses, whether made by a GP or by a psychiatrist, are entered into the patient’s primary care clinical record and are therefore recorded in the CPRD. The Read code lists that we applied to delineate PD, borderline PD, and clinically significant alcohol misuse, are available online at: https://clinicalcodes.rss.mhs.man.ac.uk [23].

### Study design and statistical analyses

The analyses were conducted using Stata v13 (Statacorps™). Due to the rarity of suicide we conducted a large nested case-control study sampled from the whole cohort at risk. The 2384 suicides in our case-control study were matched to 46,899 living controls by gender, age in years, and registered practice (median age for suicide cases and living controls = 45; age range = 16–99). Twenty controls from the same practice were selected from the age and gender-specific risk set of each case to maximise power for precise effect estimation [25]. We calculated the prevalence of PD, other mental illnesses and alcohol misuse disorder among cases and controls separately and initially generated conditional logistic regression models to estimate relative risks as exposure odds ratios (ORs) for both genders combined. These models were adjusted inherently for gender and age in the matched design. We then fitted gender interaction terms and formally tested their significance, and we estimated gender age-specific effects. For some of the analyses we also formally compared two odds ratios against each other (eg OR for PD vs. that for all other diagnosed mental illnesses; OR for borderline PD vs. that for all PD types combined).

The incidence density sampling in the nested case-control design meant that the odds ratios were interpretable as hazard ratios or incidence rate ratios, as would be derived from survival analysis of the entire cohort [26]. Although absolute risks and incidence rates cannot be estimated from a case-control design due to the sampling of living control subjects from the population at risk, the relative risk between two groups (eg people with diagnosis ‘x’ versus those without any mental disorder diagnosis) can be inferred from the exposure odds ratio value. Thus, in this hypothetical example, an observed odds ratio value of 3.0 would indicate that suicide risk was three times higher in people with diagnosis ‘x’ versus the remainder of the study population with no mental illness.

## Results

### Relative risk among all patients

These estimates are presented in Table 1. Of the 2384 suicides, 110 (4.6 %) had a Read code diagnosis of PD compared with 236 (0.5 %) of the 46,899 living controls. The exposure odds ratio indicates an almost 20-fold increased risk in these patients compared to those with no mental illness diagnosis recorded in the CPRD. The larger group of primary care patients with a different type of mental illness diagnosis had a relative risk of suicide of approximately 4.5. Direct comparison of the odds ratios between the patients with PD and those with any other mental illnesses showed that the risk was over four times greater in the PD patients, and that this was a highly significant excess risk versus other forms of mental disorder (*p* < .001).

**Table 1:** Odds ratios for suicide in patients with a personality disorder (PD) vs. no mental illness and vs. other mental illnesses

Exposure categories	Suicide cases		Living controls			
	n	%	n	%	Odds ratio^a^	(95 % CI)
No mental illness	887	37.2	33,636	71.7	1.00	(Ref.)
Other mental illness (not PD)	1387	58.2	13,027	27.8	4.47	(4.08–4.89)
Personality disorder (PD)	110	4.6	236	0.5	19.79	(15.55–25.18)
Total	2384	100	46,899	100		
PD vs. other mental illness	-	-	-	-	4.43	(3.50–5.61)

^a^Odds ratio estimated by conditional logistic regression, adjusted inherently for age and gender in the matched design

### Relative risk stratified by gender and by age

Table 2 shows relative risk estimates stratified by gender. Of the 110 suicides in total associated with PD, 75 (68.2 %) were males. A similar pattern of suicide risk was seen in both genders, with an especially large elevation in risk observed among both male and female patients diagnosed with PD. Male patients diagnosed with PD were almost 16 times more likely to die by suicide compared to patients with no mental illness, and in female patients with PD, the equivalent relative risk estimate was 38. We fitted a gender interaction term, which indicated that this large difference in effect size was statistically significant (*p* = .005). In both genders there was also a large and significant (*p* < .001) excess risk in PD patients compared to people with other mental illnesses, with the excess being 7.2 times for females compared with 3.7 times for males.

**Table 2:** Gender-specific odds ratios for suicide

Exposure categories	Suicide cases		Living controls			
	n	%	n	%	Odds ratio^a^	(95 % CI)
Men:						
No mental illness	731	40.5	26,160	74.0	1.00	(Ref.)
Other mental illness (not PD)	998	55.3	9025	25.5	4.28	(3.87–4.74)
Personality disorder (PD)	75	4.2	184	0.5	15.96	(12.02–21.19)
Total	1804		35,369			
PD vs. other mental illness	-	-	-	-	3.73	(2.82–4.93)
Women:						
No mental illness	156	26.9	7476	64.8	1.00	(Ref.)
Other mental illness (not PD)	389	67.1	4002	34.7	5.27	(4.32–6.42)
Personality disorder (PD)	35	6.0	52	0.5	38.16	(23.85–61.06)
Total	580	100	11,530			
PD vs. other mental illness	-	-	-	-	7.24	(4.63–11.34)

^a^Odds ratio estimated by conditional logistic regression, adjusted inherently for age and gender in the matched design

Similar to the gender-specific analyses described above, we then stratified the relative risk estimates into the following five age groups: 16–29, 30–39, 40–49, 50–59, 60 years and over. The youngest age band was selected to reflect young adulthood, as reported recently by the World Health Organizaton (WHO) in a suicide prevention report [27]. We then examined equal 10-year bands until 60 years and older. It was not possible to examine older age adults separately according to the 65 years and over as defined by the WHO, due to the small number of suicide cases diagnosed with PD in the case-control study sample. These results are given in Table 3. The effect sizes were significantly heterogeneous across the age strata (*p* = .01), with the greatest elevations in risk compared with those with no mental disorders being seen among the two youngest age groups (relative risks of 33 and 26, respectively). In the 60 years and older age group, the almost ten-fold increase in risk was still large and significant, but in relative terms it was a smaller elevation in risk than was observed at younger age. Again, substantial and significant (*p* < .001) excess risks compared to patients with other mental illnesses were seen across all age bands of patients with PD, with the smallest excess risk found in the older age patients.

**Table 3:** Age-stratified odds ratios for suicide

Exposure categories	Suicide cases		Living controls			
	n	%	n	%	Odds ratio^a^	(95 % CI)
16–29 years:						
No mental illness	186	51.0	5675	79.3	1.00	(Ref.)
Other mental illness (not PD)	162	44.4	1459	20.4	3.58	(2.86–4.49)
Personality disorder (PD)	17	4.7	19	0.3	32.57	(16.03–66.20)
Total	365	100	7153	100		
PD vs. other mental illness	-	-	-	-	9.09	(4.47–18.50)
30–39 years:						
No mental illness	182	33.8	7812	73.8	1.00	(Ref.)
Other mental illness (not PD)	329	61.2	2715	25.7	5.81	(4.79–7.05)
Personality disorder (PD)	27	5.0	52	0.5	25.98	(15.73–42.91)
Total	538	100	10,579	100		
PD vs. other mental illness	-	-	-	-	4.47	(2.75–7.29)
40–49 years:						
No mental illness	196	35.7	7727	71.6	1.00	(Ref.)
Other mental illness (not PD)	317	57.7	2996	27.8	4.61	(3.81–5.58)
Personality disorder (PD)	36	6.6	66	0.6	23.43	(15.13–36.28)
Total	549	100	10,789	100		
PD vs. other mental illness	-	-	-	-	5.08	(3.32–7.78)
50–59 years:						
No mental illness	125	30.0	5529	67.2	1.00	(Ref.)
Other mental illness (not PD)	274	65.7	2643	32.1	5.06	(4.04–6.34)
Personality disorder (PD)	18	4.3	52	0.6	16.81	(9.45–29.89)
Total	417	100	8224	100		
PD vs. other mental illness	-	-	-	-	3.32	(1.90–5.81)
60 years and over:						
No mental illness	198	38.5	6893	67.9	1.00	(Ref.)
Other mental illness (not PD)	305	59.2	3214	31.7	3.52	(2.91–4.25)
Personality disorder (PD)	12	2.3	47	0.5	9.46	(4.92–18.20)
Total	515	100	10,154	100		
PD vs. other mental illness	-	-	-	-	2.69	(1.41–5.15)

^a^Odds ratio estimated by conditional logistic regression, adjusted inherently for age and gender in the matched design

### Borderline PD versus all other types of PD

We examined relative risk of suicide in the small subgroup of patients diagnosed with borderline PD (Table 4). In these patients we observed a relative risk of 37 compared to patients with no mental illness, and a borderline significant doubled risk (*p* = .05) compared with all of the other patients diagnosed with PD.

**Table 4:** Odds ratios for suicide specific to patients with borderline personality disorder (BPD)

Exposure categories	Suicide cases		Living controls			
	n	%	n	%	Odds ratio^a^	(95 % CI)
No mental illness	887	37.2	33,636	71.7	1.00	(Ref.)
Mental illness (not PD or BPD)	1387	58.2	10,027	27.8	4.47	(4.08–4.89)
Other PD (not BPD)	94	3.9	217	0.5	18.30	(14.17–23.64)
Borderline PD	16	0.7	19	0.04	37.28	(19.00–73.13)
Total ^b^	2384	100	46,899	100		
BPD vs. other PD	-	-	-	-	2.04	(1.00–4.15)

^a^Odds ratio estimated by conditional logistic regression, adjusted inherently for age and gender in the matched design

^b^To avoid repetition with Table 1 we have not reported the ‘Mental illness: not PD’ in this table

### Alcohol misuse as an effect modifier

These results, which are presented in Table 5, indicate that a clinically significant level of comorbid alcohol misuse exacerbated the already heightened risk of suicide among patients diagnosed with PD. Thus, compared to patients with no mental illness or alcohol misuse diagnoses, the relative risk linked to PD alone was 16.5, whereas a markedly greater relative risk of 45 was observed in those patients diagnosed with PD and also with alcohol misuse. This excess risk in the latter subgroup of patients was statistically significant (*p* = .001).

**Table 5:** Odds ratios for suicide in patients with a personality disorder (PD) with and without a co-morbid alcohol misuse disorder

Exposure categories	Suicide cases		Living controls			
	n	%	n	%	Odds ratio^a^	(95 % CI)
No mental illness or alcohol misuse	884	37.0	33,613	71.7	1.00	(Ref.)
Mental illness (not PD or alcohol)	1234	51.8	12,494	26.6	4.15	(3.79–4.56)
Personality disorder (PD) only	80	3.4	208	0.4	16.52	(12.59–21.68)
Alcohol misuse only	156	6.5	552	1.2	12.18	(10.03–14.80)
PD + alcohol misuse	30	1.3	28	0.06	45.40	(26.84–76.78)
Total	2384	100	46,899	100		

^a^Odds ratio estimated by conditional logistic regression, adjusted inherently for age and gender in the matched design

## Discussion

### Summary of findings

Among primary care patients in CPRD, we found a 20-fold increase in risk of suicide in those diagnosed with PD compared to those with no mental disorder, and an over four-fold increase in risk when compared with patients with other mental disorder diagnoses. Females with PD had a significantly greater elevation in suicide risk than male patients with PD, compared to their gender and age-matched peers without any mental illness diagnoses. Significant risks associated with PD diagnosis were identified across all age ranges, although the greatest elevations were in the younger age ranges, 16–39 years. A diagnosis of borderline PD doubled the risk of suicide when compared to patients diagnosed with other types of PD. Clinically significant comorbid alcohol misuse significantly increased the risk of suicide in those with a diagnosis of PD.

### Comparison with previous literature

To our knowledge this is the first epidemiological study to examine suicide risk in a representative primary care sample of UK patients with PD, making it difficult to compare with other studies. However, our main findings are generally in line with published findings, except for the much larger effect sizes we found. For example, Schneider et al. [5] reported that PD was associated with a seven-fold increase in suicide risk compared with those in the general population with no mental illnesses [5]. Risks were particularly raised amongst those with a diagnosis of Cluster B personality disorders (which includes borderline type), with an odds ratio of 7.5 compared with 4.5 for Cluster A and 4.1 for Cluster C. In our study, a diagnosis of PD was associated with a 20-fold elevated risk compared with those with no mental disorders while borderline PD was linked with double the risk versus other types of PD. Schneider et al. [5] also found that being diagnosed with comorbid PD and alcohol use disorders elevated the risk 15-fold compared with those with no psychiatric diagnosis, while we found a 45-fold increase. This finding is particularly alarming, suggesting that comorbid PD and alcohol misuse represents a potent determinant of elevated suicide risk in community settings.

In a national Danish cohort study of psychiatric inpatients, Hiroeh et al. [7] reported that PD was associated with a 16-fold increase in suicide risk in women (standardised mortality ratio, SMR = 1568) and 12-fold in men (SMR = 1198) compared with people who had not been admitted for psychiatric treatment [7]. Similar patterns were found in our primary care-based study, although the effect sizes were larger still (ORs = 38 for women and 16 for men). The greater relative risk found for women may be strongly linked with the high self-harm rate in women with PD [28].

### Strengths and limitations

The CPRD uniquely provided a large, detailed, and nationally representative computerised cohort of primary care-treated patients, including those with mental illness and comorbid alcohol misuse, with complete linkage and follow-up of cause-specific mortality. Using a nested case-control design to capture all cases in the cohort at risk, and with random sampling of living matched controls from the cohort, examining risk factors for rare adverse events like suicide was possible. Biases that commonly flaw epidemiological studies can also be minimised. For example, information bias was precluded because the data were collected prospectively without knowledge of the subsequent outcome. Selection into primary care was an unlikely source of major bias because almost all UK residents are registered with a GP soon after they are born, and when people move address and register with a new practice their complete primary health care record is transferred automatically. Thus, the CPRD yields a representative community-based epidemiological sample.

The major limitation of our study was the potential for incomplete ascertainment in the CPRD of all patients diagnosed with PD, all other types of mental illness, and comorbid alcohol misuse. This is a widely recognised issue for all CPRD-based studies [17]. A second limitation concerns diagnostic validity and reliability in the Read codes that were recorded in the CPRD. All information recorded in the Datalink can be linked to a health professional within the general practice, but the link does not necessarily reflect the true diagnostic path, especially regarding information from secondary care (for example, hospital letters inputted by administrative staff). However, our study benefited from the lead author (MD) and two of the co-authors (JS and LA) having extensive clinical experience in the assessment and management of PD, thus minimising the risk of misclassification. Although we did not know how or by whom PD was diagnosed, we applied a stringent set of diagnostic codes to identify people with PD to ensure we had a clinically relevant sample. For PD to be recorded in primary care notes, it may have been diagnosed in specialist care - it is not a diagnosis commonly made by GPs and attributed to patients; thus, symptoms may be severe. Therefore, the high risk identified in the study reflects clinically evident PD rather than all patients with PD traits. Identification of ‘cases’ of clinically significant alcohol misuse was also challenging, because it is not consistently and systematically recorded in the CPRD. However, as described in the Methods section, we used a robust approach to identify alcohol misuse deemed to be at a clinically significant level. Misclassification that occurs when using the CPRD for epidemiological purposes may have attenuated the observed odds ratios conservatively towards unity because they were distributed non-differentially between suicide cases and controls [29]. A third important limitation was that we could not estimate incidence rates or absolute risk of suicide from the nested case-control design. This is true of all case-control studies, although the ‘nested’ variant with random sampling of controls from the cohort at risk is a robust statistically efficient design for examining rare adverse events such as suicide. Two final limitations of this CPRD-based study include inability to stratify PD and other mental disorders according to their degree of severity, and to examine suicide risk in patients with more than one diagnosed PD.

### Interpretation of the findings and their implications

The findings from this study highlight the importance of considering age, gender and concurrent alcohol misuse when assessing suicide risk among primary care patients diagnosed with PD. With increasing age, certain risky behaviours associated with PD and suicide, such as impulsivity and aggression, may become less common, contributing to the lower elevated suicide risks amongst those from the older age groups [30]. However, comorbid psychiatric disorders may enhance the expression of other dysfunctional traits such as dementia and social withdrawal in older people making it more complex to diagnose PD in this group [30, 31]. The diagnostic criteria for PD and in particular borderline PD - including repeated non-fatal self-harm, impulsivity, affective instability, and mood disorder - have consistently been found to be strongly linked to suicidal behaviour [2, 4, 32, 33]. Self-harming behaviour among people with a diagnosis of PD, but especially borderline PD, tends to be ambivalent in intent where emotional instability and impulsivity are very common. This is complicated further as often the level of intent of self-harm can fluctuate rapidly and trying to accurately predict a rare event such as suicide is seldom possible. Therefore, enhancing the skills of GPs, through training opportunities, to identify and assess PD and personality traits factors could help in reducing the risk of suicide; this would need to take account of the high false positive rate amongst those who repeatedly self-harm. A standardised assessment tools such as the Standardised Assessment of Personality [34] may be helpful as a first-stage screen as part of a two-stage procedure for case identification [35], which could be used regardless of the age or gender of patients and could be integrated into a primary care consultation.

Similar to mental health services, it is possible that the elevated risk of suicide in primary care is related to a paucity of effective interventions to manage the risk. There is a lack of consensus about the extent to which people with PD are amenable to therapeutic interventions [36] and primary health care staff may view people with PD as ‘untreatable’. A sense of futility combined with a lack of therapeutic skills could be exacerbating an already elevated risk among these patients. Closely linked to perceived ‘untreatabilty’ is the tendency for patients with PD to be viewed negatively by treating psychiatrists and other clinicians due to their antisocial, disruptive and treatment resistant symptoms [37], especially if they self-harm [38]. There is also some evidence that those with a diagnosis of PD are less likely than those without to be referred by GPs to specialist psychiatric services [14] and GPs have been shown to view those with a PD as ‘less compliant’, ‘less likeable’ and ‘more stressful’ to deal with [14]. This may be further exacerbated by time-limited consultations.

Nevertheless, GPs remain in a unique position to assess the risk of suicide in people with PD and to intervene. This would mean working with specialist care, agreed clinical pathways and availability of services for comorbidities such as alcohol misuse, as well as opportunities for GPs to develop specific clinical skills. Presence of PD traits, alcohol misuse and suicidal behaviour and frequent attendance at clinic could be the basis for a ‘flag’ alert in primary care records for treatment and/or referral [16]. Given that a diagnosis of alcohol use disorders is associated with a much heightened risk of suicide in patients with PD, detection and treatment for alcohol misuse should be a priority [39]. In addition, since impulsivity is a trait that is common among people diagnosed with PD, substance use and suicidal behaviour [32, 40] treatments should also target difficulties in impulse control.

Given the strong link found in this study between PD and suicide in primary care, and the paucity of robust evidence in this setting, there is a clear need for further research. Attempts should be made to try and replicate the findings from this study in different primary care samples from other countries. From a clinical perspective, the way that GPs assess, support and manage people with PD should be further explored, both quantitatively and qualitatively. A review of the links between primary and specialist mental health services would be useful to establish the mutual expectations of the referrer and referee and to evaluate treatment options available [41]. The role of specialist mental health services and the interface with primary care services in managing the needs and risks among people with PD also requires evaluation.

## Conclusions

In a large representative sample of UK primary care patients, we found elevated risk of suicide amongst those with PD versus those with no recorded mental disorder or other psychiatric illnesses. The risks were particularly raised amongst those with comorbid alcohol misuse disorder or borderline PD. GPs have a potentially key role to play in reducing risk of suicidal behaviour by intervening with patients diagnosed with PD, particularly when comorbid alcohol misuse is present.

## Abbreviations

BPD, borderline personality disorder; CPRD, Clinical Practice Research Datalink; GP, general practitioner; NHS, National Health Service; ONS, Office for National Statistics; OR, odds ratio; PD, personality disorder; RAG, Red/Amber/Green; SMR, standardised mortality ratio; WHO, World Health Organizaton

## References

[1] ONS. What are the top causes of death by age and gender? February 27, 2015. http://visual.ons.gov.uk/what-are-the-top-causes-of-death-by-age-and-gender. Accessed 17 Nov 2015

[2] Black DW, Blum N, Pfohl B, Hale N (2004). Suicidal behavior in borderline personality disorder: Prevalence, risk factors, prediction, and prevention. J Pers Disord.

[3] Paris J, Zweig-Frank H (2001). A 27-year follow-up of patients with borderline personality disorder. Compr Psychiatry.

[4] Samuels J (2011). Personality disorders: Epidemiology and public health issues. Int Rev Psychiatry.

[5] Schneider B, Schnabel A, Wetterling T, Bartusch B, Weber B, Georgi K (2008). How do personality disorders modify suicide risk?. J Pers Disord.

[6] Baxter D, Appleby L (1999). Case register study of suicide risk in mental disorders. Br J Psychiatry.

[7] Hiroeh U, Appleby L, Mortensen PB, Dunn G (2001). Death by homicide, suicide and other unnatural causes in people with mental illness: a population-based study. Lancet.

[8] Kapur N, Hunt I, Windfuhr K, Rodway C, Webb R, Rahman M (2013). Psychiatric in-patient care and suicide in England, 1997 to 2008: A longitudinal study. Psychol Med.

[9] Krysinska K, Heller TS, De Leo D (2006). Suicide and deliberate self-harm in personality disorders. Curr Opin Psychiatry.

[10] Soloff PH, Lynch KG, Kelly TM, Malone KM, Mann JJ (2000). Characteristics of suicide attempts of patients with major depressive episode and borderline personality disorder: a comparative study. Am J Psychiatry.

[11] Agrawal A, Narayanan G, Oltmanns TF (2013). Personality pathology and alcohol dependence at midlife in a community sample. Personal Disord.

[12] Goldstein RB, Dawson DA, Chou SP, Grant BF (2012). Sex differences in prevalence and comorbidity of alcohol and drug use disorders: Results from wave 2 of the National Epidemiologic Survey on Alcohol and Related Conditions. J Stud Alcohol Drugs.

[13] Trull TJ, Jahng S, Tomko RL, Wood PK, Sher KJ (2010). Revised NESARC personality disorder diagnoses: gender, prevalence, and comorbidity with substance dependence disorders. J Pers Disord.

[14] Moran P, Rendu A, Jenkins R, Tylee A, Mann A (2001). The impact of personality disorder in UK primary care: a 1-year follow-up of attenders. Psychol Med.

[15] NCISH (2015). National confidential inquiry into suicide and homicide by people with mental illness. Annual report: England, Northern Ireland, Scotland and Wales.

[16] NCISH (2014). Suicide in primary care in England: 2002–2011.

[17] Herrett E, Gallagher AM, Bhaskaran K, Forbes H, Mathur R, van Staa T (2015). Data Resource Profile: Clinical Practice Research Datalink (CPRD). Int J Epidemiol.

[18] Herrett E, Thomas SL, Schoonen WM, Smeeth L, Hall AJ (2010). Validation and validity of diagnosis in the General Practice Research Database: a systematic review. Br J Clin Pharmacol.

[19] Khan NF, Harrison SE, Rose PW (2010). Validity of diagnostic coding within the General Practice Research Database: a systematic review. Br J Gen Pract.

[20] Adelstein A, Mardon C (1975). Suicides 1961–1974.

[21] Linsley K, Schapira K, Kelly T (2001). Open verdict v suicide – importance to research. Br J Psychiatry.

[22] Olier I, Springate DA, Ashcroft DM, Doran T, Reeves D, Planner C (2016). Modelling conditions and health care processes in electronic health records: an application to severe mental illness with the Clinical Practice Research Datalink. PLoS One.

[23] Springate DA, Kontopantelis E, Ashcroft DM, Olier I, Parisi R, Chamapiwa E (2014). Clinical Codes: an online clinical codes repository to improve the validity and reproducibility of research using electronic medical records. PLoS One.

[24] Harris EC, Barraclough B (1997). Suicide as an outcome for mental disorders. A meta-analysis. Br J Psychiatry.

[25] Hennessy S, Bilker WB, Berlin JA, Strom BL (1999). Factors influencing the optimal control-to-case ratio in matched case–control studies. Am J Epidemiol.

[26] Clayton M, Hills D (1993). Statistical models in epidemiology.

[27] World Health Organization (2014). Preventing suicide: a global imperative.

[28] Hawton K (2000). Sex and suicide: Gender differences in suicidal behaviour. Br J Psychiatry.

[29] Copeland KT, Checkoway H, McMichael AJ, Holbrook RH (1977). Bias due to misclassification in the estimation of relative risk. Am J Epidemiol.

[30] Mordekar A, Spence S (2008). Personality disorder in older people: how common is it and what can be done?. Adv Psychiatr Treat.

[31] Devanand DP (2002). Comorbid psychiatric disorders in late life. Biol Psychiatry.

[32] Brodsky BS, Malone KM, Ellis SP, Dulit RA, Mann JJ (1997). Characteristics of borderline personality disorder associated with suicidal behavior. Am J Psychiatry.

[33] Yen S, Shea MT, Sanislow CA, Grilo CM, Skodol AE, Gunderson JG (2004). Borderline personality disorder criteria associated with prospectively observed suicidal behavior. Am J Psychiatry.

[34] Moran P, Leese M, Lee T, Walters P, Thornicroft G, Mann A (2003). Standardised Assessment of Personality – Abbreviated Scale (SAPAS): preliminary validation of a brief screen for personality disorder. Br J Psychiatry.

[35] Lenzenweger MF, Loranger AW, Korfine L, Neff C (1997). Detecting personality disorders in a nonclinical population. Application of a 2-stage procedure for case identification. Arch Gen Psychiatry.

[36] Bateman AW, Gunderson J, Mulder R (2015). Treatment of personality disorder. Lancet.

[37] Lewis G, Appleby L (1988). Personality disorder: the patients psychiatrists dislike. Br J Psychiatry.

[38] Rayner G, Allen S, Johnson M (2005). Countertransference and self-injury: a cognitive behavioural cycle. J Adv Nurs.

[39] Links PS, Heslegrave RJ, Mitton JE, van Reekum R, Patrick J (1995). Borderline personality disorder and substance abuse: consequences of comorbidity. Can J Psychiatry.

[40] Bornovalova MA, Lejuez CW, Daughters SB, Rosenthal MZ, Lynch TR (2005). Impulsivity as a common process across borderline personality and substance use disorders. Clin Psychol Rev.

[41] Chew-Graham CA, Slade M, Montana C, Stewart M, Gask L (2008). The loss of doctor-to-doctor communication: Lessons from the reconfiguration of mental health. J Health Serv Res Policy.

